# Impact of Sequential Chemical Doping on the Thin Film Mechanical Properties of Conjugated Polymers

**DOI:** 10.1021/acs.chemmater.4c03120

**Published:** 2025-01-09

**Authors:** Kan Tang, Alyssa Shaw, Saroj Upreti, Haoyu Zhao, Yunfei Wang, Gage T. Mason, Jeffrey Aguinaga, Keyi Guo, Derek Patton, Derya Baran, Simon Rondeau-Gagné, Xiaodan Gu

**Affiliations:** † Center for Optoelectronic Materials and Devices, School of Polymer Science and Engineering, The University of Southern Mississippi, Hattiesburg, Mississippi 39406, United States; ‡ Materials Science and Engineering Program (MSE), Physical Sciences and Engineering Division (PSE), King Abdullah University of Science and Technology, Thuwal 23955, Saudi Arabia; § Department of Chemistry and Biochemistry, University of Windsor, Windsor, ON N9B 3P4, Canada; ∥ Oak Grove High School, Hattiesburg, Mississippi 39402, United States

## Abstract

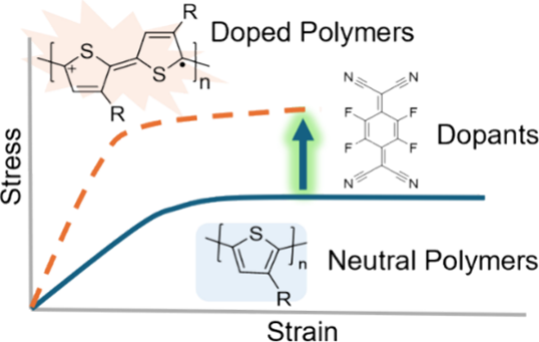

Conjugated polymer (CP) films with nanometer-scale thickness exhibit unique properties distinct from their bulk counterparts, which is an important consideration for their end application as thin film devices. In the realm of organic electronic devices, enabling high electrical conductance properties of CPs often necessitates doping. However, the impact of doping on intrinsic polymer mechanical properties, such as the elastic modulus, in ultrathin films at device-relevant thicknesses is not well understood and has not been directly measured. In this study, we quantified the effect of doping on the mechanical properties of poly(3-alkylthiophenes) (P3ATs) using pseudofree-standing tensile testing. We observed modulation of the mechanical properties of ultrathin CP films through sequential doping of P3ATs thin films (60–80 nm thick) with the molecular dopant F4TCNQ. Our findings reveal that, despite the ease of doping all P3ATs with F4TCNQ, the resulting changes in mechanical properties are highly dependent on the side-chain lengths of the P3ATs. Specifically, the elastic modulus of rubbery P3ATs with side-chain lengths of six carbons or more (e.g., P3HT and P3OT) increases significantly—by one to two times—upon F4TCNQ doping, while the modulus of the glassy poly(3-butylthiophene-2,5-diyl) (P3BT) remains nearly unchanged. Such a phenomenon is linked to the changes in the glass transition temperature (*T*_g_) of the doped film, where the rise of *T*_g_ results in a large change in the modulus for P3HT samples. However, the P3BT remained in a glassy state before and after doping, exhibiting a minimal change in its mechanical properties. These insights into the mechanical behavior of doped ultrathin CP films are crucial for the design and optimization of flexible electronic devices.

## Introduction

Conjugated polymers (CPs) offer advantages such as flexibility, chemical tunability, and solution processability, making them increasingly attractive for applications in devices like organic field effect transistors (OFETs),^1−4^ organic solar cells (OSCs),^5,6^ thermoelectric devices, bioelectronics, and stretchable electronics.^7−10^ While OFETs and OSCs primarily rely on the semiconducting properties of CPs, several applications require CPs to exhibit high electrical conductivity. In their pristine or nondoped state, CPs often lack sufficient conductivity to meet the demands of certain applications, such as organic electrodes. To enhance the conductivity of CPs, molecular doping is used to either oxidize (p-doping) or reduce (n-doping)^11^ the polymers, thereby increasing the concentration of charge carriers. For example, blending CPs with dopants can improve OFETs performance by passivating traps^12−14^ and modifying threshold voltages.^15,16^ Chemical doping also serves as a straightforward method to produce high-performance organic thermoelectric devices that require high electrical conductivity (σ > 1 S/cm), since conductivity is proportional to the power factor (α^2^σ) of the device.^17−19^ Additionally, the doping level of CPs can also be controlled electrochemically, as demonstrated in emerging organic electrochemical transistors (OECTs).^20^ In a typical depletion mode p-type OECT devices using thiophene-based materials as P3HT,^21,22^ P(g2T-T/TT)^23−25^ or PEDOT:PSS,^26,27^ the doping level decreases under a positive gate bias and increases upon a negative gate bias, allowing for control over the channel current. In all these cases, the critical role of doping in influencing the electrical performance and optical properties of polymeric thin film devices has been thoroughly investigated and understood. However, there is a relative lack of understanding regarding the mechanical properties of doped films, especially those thinner than 100 nm, which could lead to unforeseen issues when employing doped CPs in flexible electronics. Unintended changes in the mechanical properties can result in a mismatch between different layers and cause cracks or delamination upon deformation. Early work by Heeger’s group shows a reduction in the elastic modulus (*E*) of *in situ* polymerized films of polyacetylene^28^ or fibers of poly(2,5-dimethoxy-*p*-phenylenevinylene)^29^ upon doping by iodine vapor, decreasing from tens of GPa by typically a factor of approximately five. Conversely, several folds increase in modulus can be observed in iodine-doped polymers such as poly(2,5-thienylene vinylene) fibers.^30^ However, the opposite modulation in the elastic modulus observed in different CPs is not well understood at the time. Müller’s group explored the mechanical properties of doped, thiophene-based polymers using bulk tensile testing through dynamic mechanical analysis (DMA) and discovered similar trends in the modulation of elastic modulus.^31−33^ By surveying a series of dopants such as Mo(tfd-COCF_3_),^34^ F4TCNQ,^33,34^ or EBSA,^35^ they found that the elastic modulus of CPs decreases upon doping when the neutral polymer is stiff (i.e., *E* > 100 MPa) such as P3HT^34−36^ and increases upon doping if the neat polymer is “soft” (i.e., *E* < 100 MPa) such as P3OT and P(g2T-T).^33,37,38^ The observed softening in stiff polymers is attributed to the plasticization effect introduced by the addition of the large amount of dopant,^32,33^ whereas, the stiffening in soft polymers results from an increase in crystallinity after chemical doping.^37,38^ For a comprehensive summary of the impact of doping on the mechanical properties of bulk CPs, refer to the recent review by Müller’s group.^39^

Although doped CP films derived from *in situ* polymerization,^28^ drop-casting,^32−34^ or hot-press,^35^ have been successfully characterized, the resulting bulk tensile samples typically have thicknesses of tens of micrometers. Since nanoscale thin films are prevalent in semiconducting devices, gaining a deeper understanding of the relationship between doping and the mechanical response of CP films thinner than 100 nm is essential for optimizing thin-film mechanics in wearable devices. Furthermore, thick (∼100 μm) doped samples prepared from poorly soluble CP/dopant mixtures^40,41^ or vapor doping^28,33^ may compromise the homogeneity of dopant distribution and surface roughness, making them less ideal for mimicking real devices. Our group has previously developed a thin-film tensile testing method for measuring the mechanical properties of CP films on water. For those interested in a more in-depth explanation of this technique, we have published a review article on this topic together with a few publications.^42−46^ Up to now, such a thin film tensile technique has not been applied to doped CPs.

**Figure 1:**
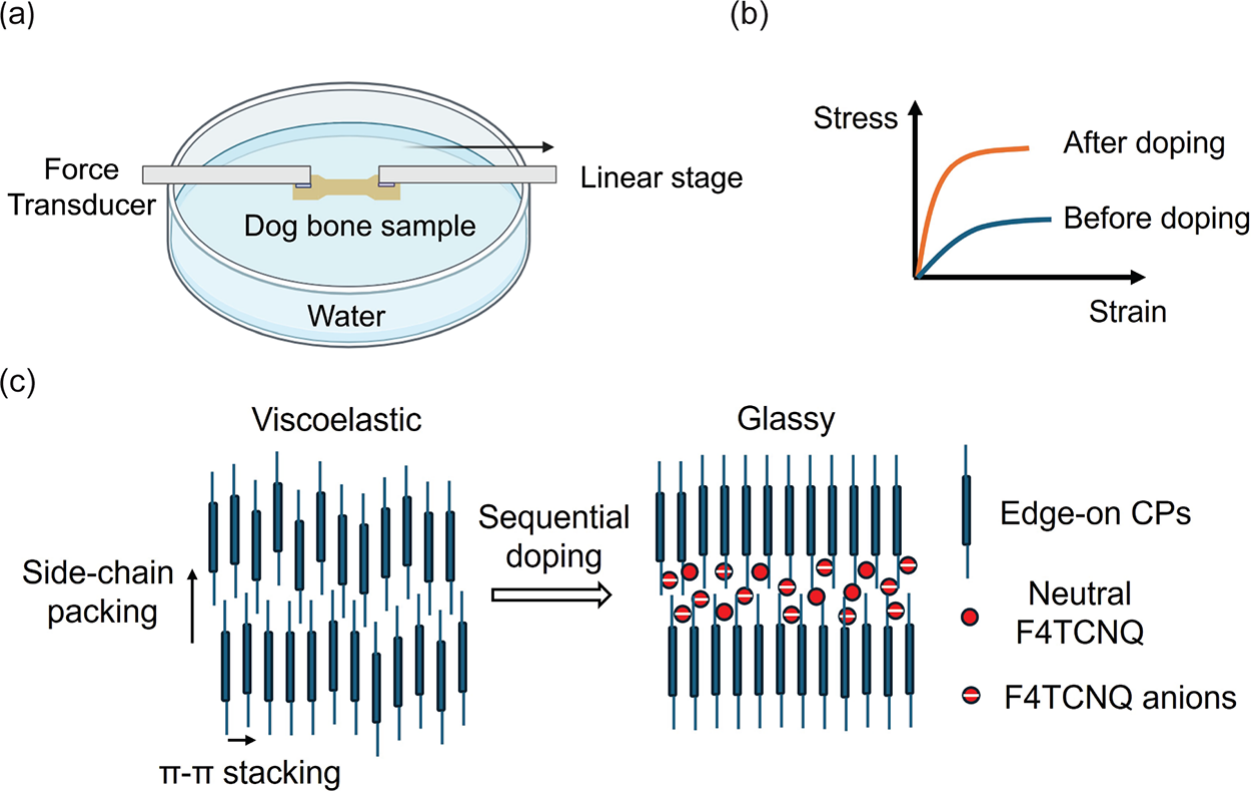
(a) Schematic illustration of the pseudofree-standing tensile tests setup. The film-on-water dog-bone tensile sample is mounted by two separate PDMS grips from a force transducer and a linear stage. (b) Characteristics of the stress–strain relationship of pristine (blue line, viscoelastic) and doped (red line, glassy) CPs. (c) Schematic representation of the sequential doping process of CPs via infiltration of the dopants into the edge-on CP matrix as an example. As suggested by the previous report,^47−49^ F4TCNQ tends to intercalate into the side-chain packings.

Here, we studied the mechanical properties of sub-100 nm doped polythiophene and poly(diketopyrrolopyrrole-*co*-thiophene) (DPP-T) films by using the pseudofree-standing tensile test method (Figure 1a) and obtained full stress–strain relationships of pristine and doped CP thin films floating on water. We employed moderate sequential doping (Figure 1c) to minimize the impact on film roughness as the dopant preferably diffuses into the lamellar region of the CPs such as P3HT during the sequential doping process,^47^ enabling high-quality films for thin film tensile testing. Our findings reveal that the enhancement of the elastic modulus as illustrated schematically (Figure 1b) in F4TCNQ-doped P3AT thin films is influenced by the side-chain length of the CP and its glass transition temperature (*T*_g_). Viscoelastic P3HT and P3OT exhibit a 1- to 2-fold increase in elastic modulus after doping, while the modulus of the glassy P3BT remains nearly unchanged. Beyond the P3AT system, we also investigated the effect of doping on the thin film mechanics of the DPP-T polymer. The introduction of the F4TCNQ dopant into a sub-100 nm DPP-T polymer did not result in effective doping due to an energetic mismatch between the polymer and the dopant. Consequently, no change in the elastic modulus of the samples was observed, indicating a negligible plasticizing effect from the F4TCNQ dopant. These observations suggest that the efficiency of doping and the dynamics of the pristine polymer prior to doping are crucial factors influencing the final modulus.

## Results

**Figure 2:**
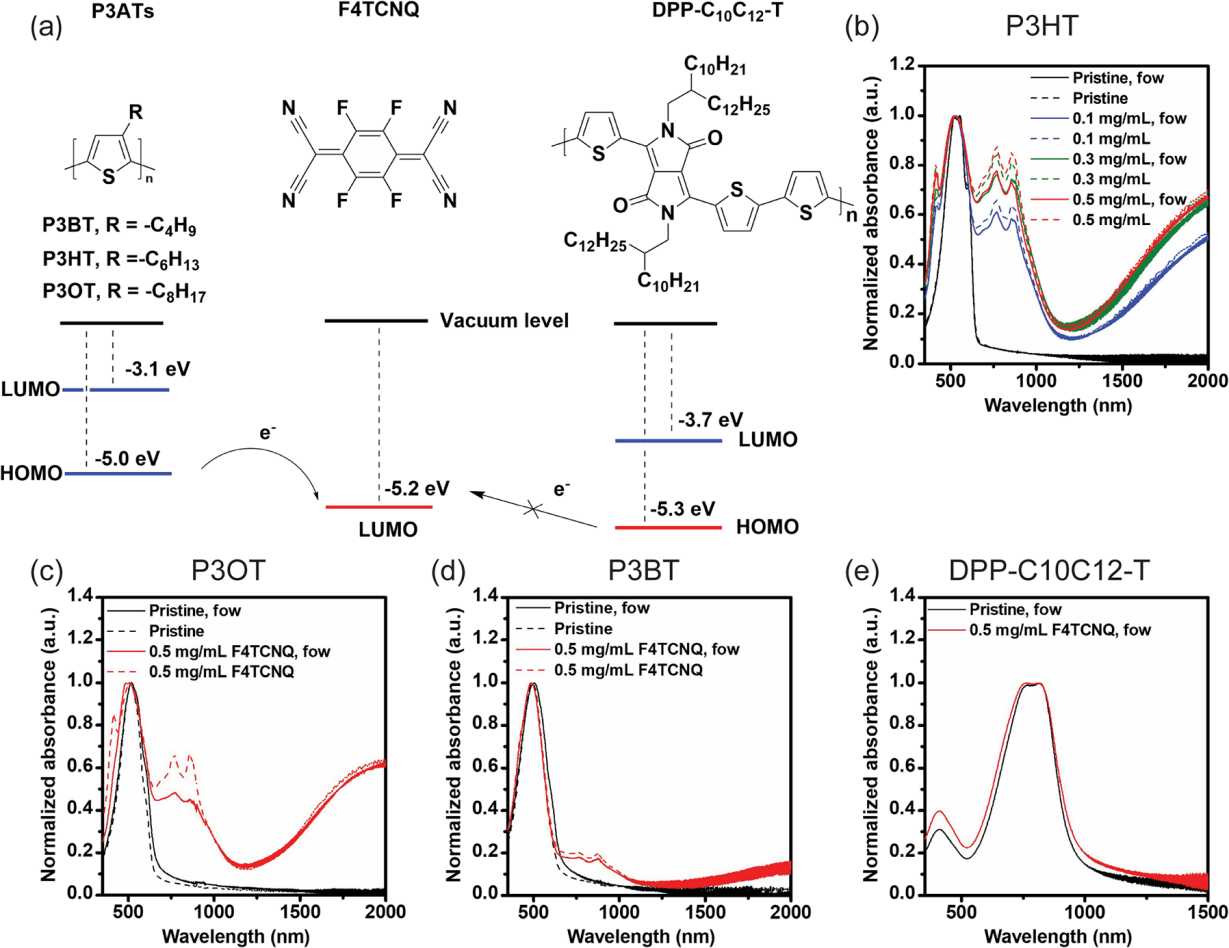
(a) Chemical structures and energetics of P3ATs and F4TCNQ dopant. As examples, HOMO and LUMO levels of P3HT,^50^ F4TCNQ,^51^ and DPP-C_10_C_12_-T^52^ were obtained from the literature. (b–e) UV–vis spectra of pristine and sequentially doped P3HT, P3OT, P3BT, and DPP-C_10_C_12_-T thin films. The dashed lines represent spectra of P3ATs/PSS/glass samples, and the solid lines are associated with film-on-water samples. (b) P3HT films doped with 0.1, 0.3, and 0.5 mg/mL of FT4CNQ in acetonitrile (F4TCNQ/AN). (c) P3OT films doped with 0.5 mg/mL F4TCNQ/AN. (d) P3BT films doped with 0.5 mg/mL F4TCNQ/AN. (e) DPP-C_10_C_12_-T films doped with 0.5 mg/mL F4TCNQ/AN. Note that 0.5 mg/mL F4TCNQ/AN is equivalent to 1.1 mM.

**Figure 3:**
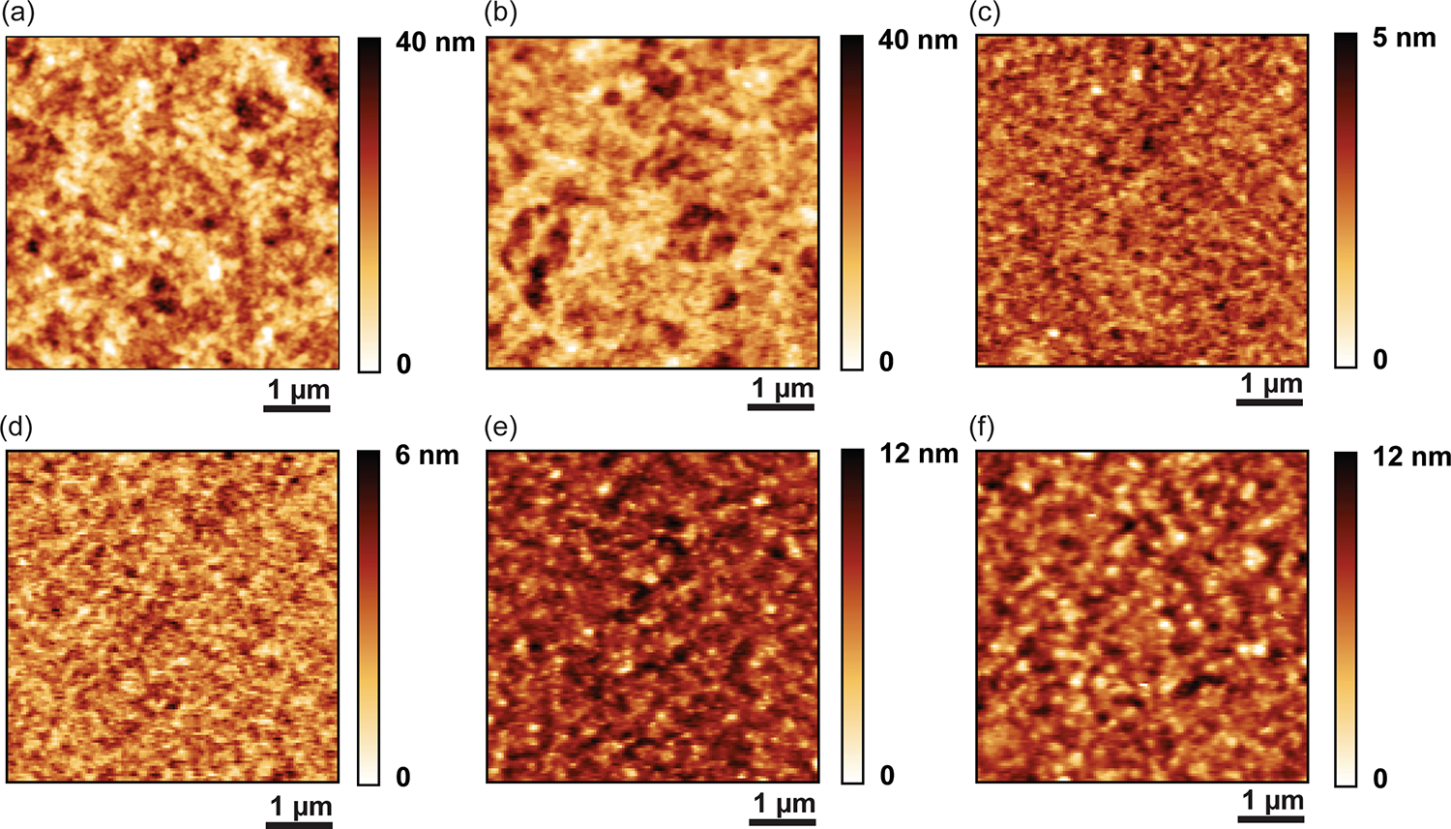
AFM images of pristine and doped P3ATs. (a) Pristine P3HT. (b) P3HT doped with 0.5 mg/mL F4TCNQ/AN. (c) Pristine P3OT. (d) P3OT doped with 0.5 mg/mL F4TCNQ/AN. (e) Pristine P3BT. (f) P3BT doped with 0.5 mg/mL F4TCNQ/AN. All images are in a size of 5 × 5 μm square.

The mechanical response of the doped thin film CPs (Figure 2a) is probed by the film-on-water (FOW) technique. However, successful FOW tensile testing requires films with high uniformity to ensure that surface roughness does not affect the interpretation of the elastic modulus.^42,43^ Two common methods for doping CP thin films are spin-casting from a doped solution and sequential doping. Previous studies have reported that doping CPs in solution leads to the formation of poorly solubilized polarons and anionic products.^40,41^ Therefore, the roughness of doped spin-coated films from solution mixing can be as high as several tens of nm.^41^ On the other hand, sequential doping has been proposed to overcome the surface uniformity issue associated with the solution doping method.^40,41^ In this approach, a dopant solution dissolved in an orthogonal polar solvent like acetonitrile (AN) is spin-coated onto the predeposited CP layer. It has been shown that by carefully selecting the concentration of the dopant solution, one can achieve effective chemical doping while preserving the uniformity of the film.^40,53^Figure 3 presents the surface morphology of both pristine and F4TCNQ-doped films as observed through tapping-mode Atomic Force Microscopy imaging. Little to no alteration in morphology is noticeable between the pristine films (Figure 3a,c,e) and the doped films (Figure 3b,d,f) for each CP. The surface roughness (RMS) of P3HT remains at 6 nm after sequential doping (Table S5, entries 1–4). Similarly, P3OT and P3BT exhibit minimal changes in RMS values after doping, with differences less than 0.6 nm (Table S5, entries 8–9 and 11–12). All the sequentially doped samples maintained low RMS values both before and after doping, which are significantly smaller than the typical film thickness (60–80 nm), thereby satisfying the film quality requirements for a pseudofree-standing tensile test. Thus, the sequential doping method was used to prepare suitable samples for FOW tensile tests.

Figure 2b–d presents the UV–vis spectra of doped and undoped films, illustrating the effectiveness of the sequentially doped thin CP films. In general, F4TCNQ (EA = −5.2 eV^51^) can readily dope P3ATs (i.e., IE_P3HT_ = −5.0 eV^50^), as electron transfer from P3ATs to the dopant is energetically favored. In the sequentially doped P3ATs samples (dashed lines in Figure 2b–d), the signature polaron absorption band of P1 (>1200 nm) and P2 (700–1000 nm) is evident in all P3ATs,^54,55^ along with two adjacent peaks corresponding to F4TCNQ anions at 768 and 860 nm.^54,55^ As the F4TCNQ dopant concentration increases from 0.1 mg/mL to 0.5 mg/mL (Figure 2b), the intensities of both the P3HT polaron band and the anion peaks increase correspondingly, indicating that higher doping levels are attainable with higher dopant concentrations. The doping efficiency can be estimated by the ratio of the peak intensities between the F4TCNQ anion (768 nm) and neutral P3AT (530 nm). Doping efficiency is highest in P3HT as *I*_768 nm_/*I*_500 nm_ ∼ 0.85, while the efficiency is lower in P3OT (∼0.65) and P3BT (∼0.2).

Since our FOW tensile testing involves exposing samples to a water surface, we examined the effect of water on the doping status of the doped CPs by spectroscopically characterizing the samples before and after water exposure. The UV–vis spectra of samples after water exposure retained the distinctive absorption features (solid lines Figure 2b–d) of doping that resemble those in the doped P3AT samples with water exposure. However, the absorption intensities of F4TCNQ anion (768 and 860 nm) decreased compared to the unexposed samples. Previously, Watt et al. reported the transformation of F4TCNQ anion/dianion into HF4TCNQ^–^ species upon exposure to the protic environment such as acids or water.^56^ Therefore, the reduction in F4TCNQ anion absorption can be attributed to the interconversion of the anion into hydrogen-adducted products. Interestingly, the polaron bands (P1 and P2) showed minimal decay, indicating the polarons are not dramatically affected by floating films on water. Additionally, X-ray photoelectron spectroscopy (XPS) probes on N_1s_ and F_1s_ core electrons remained almost constant (∼3.8 mol % *v.s.* C_1s_, Tables S1 and S2) before and after floating doped films on the water for 10 min (Figure S1a,b). This observation supports the preservation of the doped status without significant leaching of dopants or dedoping of the films. Despite the conversion of the F4TCNQ anion into other anionic species, the doping associated with polaron backbone structures of the CPs is preserved for the doped FOW samples. This ensures that we can measure the mechanical properties of the F4TCNQ-doped films on a water surface.

**Figure 4:**
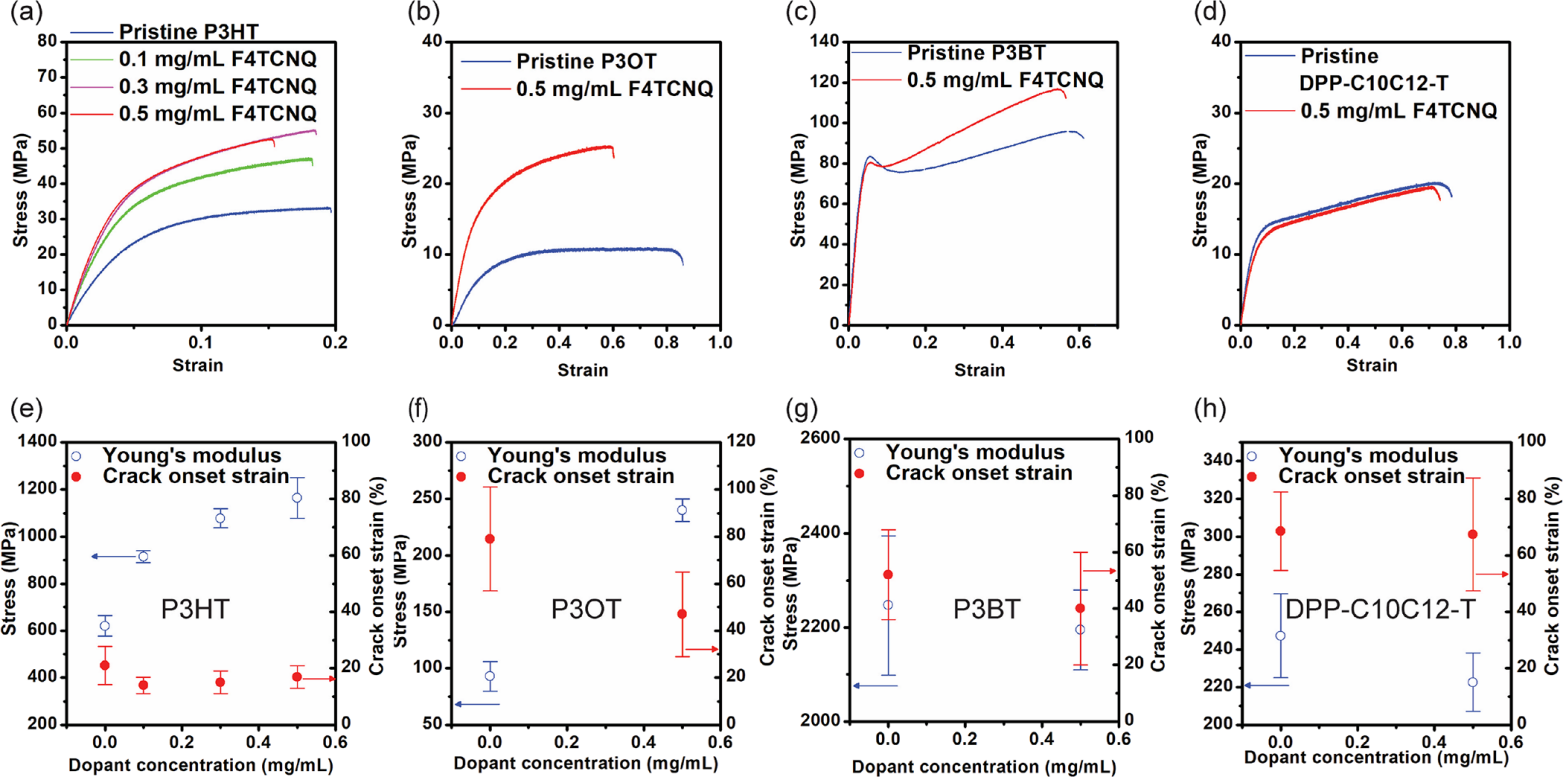
(a–d) Stress–strain curves of pristine and F4TCNQ-doped CPs using the pseudofree-standing tensile test. (a) P3HT. (b) P3OT. (c) P3BT. (d) DPP-C_10_C_12_-T. (e–h) Stress and strain dependence of the corresponding CPs on the F4TCNQ dopant concentrations.

Next, we used FOW tensile testing to investigate the mechanical properties of sequentially doped P3ATs and DPP polymers with film thickness under 100 nm. Full stress–strain curves for both pristine and sequentially doped P3AT films are presented in Figure 4a–c. Doping the thin film with an F4TCNQ concentration of 0.1 mg/mL resulted in a 48% increase in elastic modulus, from approximately 600 to 900 MPa. The film can be further stiffened by using higher dopant concentrations. At 0.5 mg/mL dopant concentration, the modulus doubles compared to the undoped state, exceeding 1200 MPa (Figure 4e). To confirm the durability of the doping effect on mechanical properties, we conducted stress relaxation experiments. As illustrated in Figure S2, both the doped and undoped samples showed stress relaxation. This is ascribed to the stress relaxation mostly occurring at the phase-separated side-chain region. Even after doping, the side chains are still viscoelastic domains and allow stress to dissipate.^42,57^ In contrast to the approximate doubling of the elastic modulus, the change in crack onset strain between pristine and doped films is less pronounced. The ductility of the doped P3HT films decreases by 20–30% in crack onset strain after doping with various concentrations of F4TCNQ in acetonitrile. A similar stiffening effect induced by doping is also observed in P3OT. As shown in Figure 4f, the elastic modulus of P3OT increases from 93 to 240 MPa after sequential doping with 0.5 mg/mL F4TCNQ/AN, accompanied by a reduction in crack onset strain. In addition to regioregular (rr) P3ATs, the mechanical properties of regiorandom (rra) polythiophene before and after F4TCNQ doping were also investigated. The modulus of rra-P3HT increases from 12 to 33 MPa upon sequential doping with F4TCNQ, while the crack onset strain reduces from 530% to 350% (Figure S3). Therefore, the observed trend of enhanced modulus and lowered crack onset strain of viscoelastic P3ATs (P3HT, P3OT) upon F4TCNQ doping is independent of the regioregularity. However, in the case of P3BT (Figure 4g), there is no noticeable increase in elastic modulus; it remains around 2300 MPa after sequential doping.

In addition to measuring the mechanical properties of doped P3ATs, we studied the doping effect on a representative donor–acceptor (D–A) type conjugated polymer, DPP-C_10_C_12_-T. As depicted in Figure 2e, the spectrum of the F4TCNQ-treated DPP-C_10_C_12_-T floating-on-water (FOW) sample closely overlaps with that of the pristine polymer, showing no noticeable new absorption bands or peaks from doping products. Unlike the facile doping observed in the previously discussed P3ATs, the absence of such features confirms the much lower doping efficiency in DPP-T type D–A polymers. Consequently, the mechanical properties of the F4TCNQ-treated DPP-C_10_C_12_-T exhibit negligible differences in the stress–strain curve (Figure 4d), indicating that F4TCNQ does not modulate the mechanical properties (Figure 4h) of DPP-C_10_C_12_-T thin films.

## Discussion

**Figure 5:**
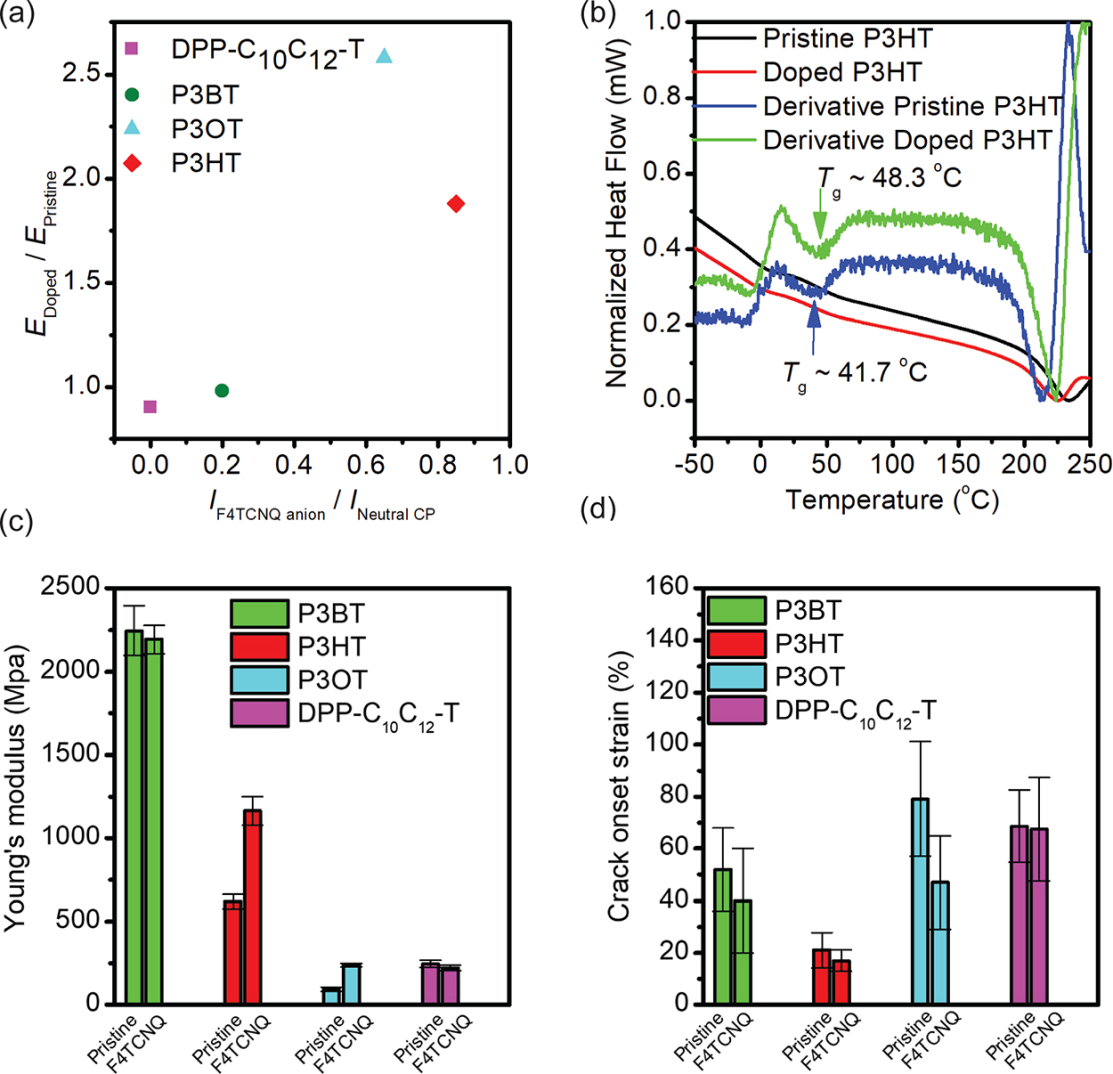
(a) Enhancement of the elastic modulus (*E*_doped_/*E*_pristine_) of CPs upon sequential doping by F4TCNQ/AN as a function of doping efficiency. The doping efficiency of a CP/dopant pair is approximated by the UV–vis absorbance ratio of a doping product peak (*I*_F4TCNQ anion_) to its neutral CP peak (*I*_neutral CP_). (b) Flash DSC thermogram of the drop-cast films of F4TCNQ/AN-treated P3HT (red line) and pristine P3HT (black line). (c) Summary of the elastic modulus (*E*) of pristine/doped CPs. (d) Summary of the crack onset strains of pristine/doped CPs.

Figure 5c,d summarizes the modulation of mechanical properties for all the CPs studied in this work through F4TCNQ doping or treatment. A 1 to 2-fold increase in elastic modulus is observed in F4TCNQ-doped P3HT and P3OT, while P3BT and DPP-C_10_C_12_-T exhibit little change in modulus after doping. Doping with F4TCNQ leads to less ductile thin films of P3ATs, as indicated by lower COS, whereas the crack onset strain of DPP-C_10_C_12_-T remains unchanged. The F4TCNQ-doped/treated thin films either become “stiffened” or maintain their original modulus. Overall, the modulation of mechanical properties in thin-film tensile tests of CPs by sequential F4TCNQ doping is highly dependent on the physical state of the sample—viscoelastic or glassy—which is strongly influenced by the side-chain length, as well as on doping efficiency (e.g., charge transfer energetics). P3HT and P3OT, with longer side chains, have *T*_g_s that are lower or close to room temperature, making them viscoelastic polymers.^58^ Doping of those CPs by F4TCNQ could result in “frozen” backbones that cause an observable stiffening effect of the doped film. In contrast, P3BT has a much higher *T*_g_ of 45 °C, meaning it is already in a glassy state at room temperature.^58^ After doping, the sample is again in the glassy region. Therefore, its modulus is insensitive to additional stiffening through sequential doping, and we observe a much lower doping efficiency compared to P3AT samples with shorter side chains (Figure 5a). Regarding D–A polymers like DPP-C_10_C_12_-T, due to their low laying HUMO levels (IE < −5.2 eV),^52,59^ the doping efficiency is much lower than a typical thiophene-based conjugated polymer. Efficient doping has been reported only when using highly oxidative dopants such as F6TCNNQ^60^ (EA = −5.37 eV) or CN6-CP^61^ (EA = −5.87 eV). Thus, despite its low *T*_g_ of ∼ −4 °C in pristine form,^58^ the mechanical properties of DPP-C_10_C_12_-T as a viscoelastic polymer are unchanged due to insufficient doping of the polymers by F4TCNQ. No significant plasticizing effect was found in the CPs doped/treated with hydrophobic F4TCNQ dopant in all cases discussed above. However, despite facile doping by hydrophilic FeCl_3_ dopant (Figure S4), the plasticizing effect is evident by a drop in the elastic modulus of P3HT and P3BT as shown in Figures S5 and S6. Details on the FeCl_3_ doping experiments can be found in Section 9 of the Supporting Information.

**Figure 6:**
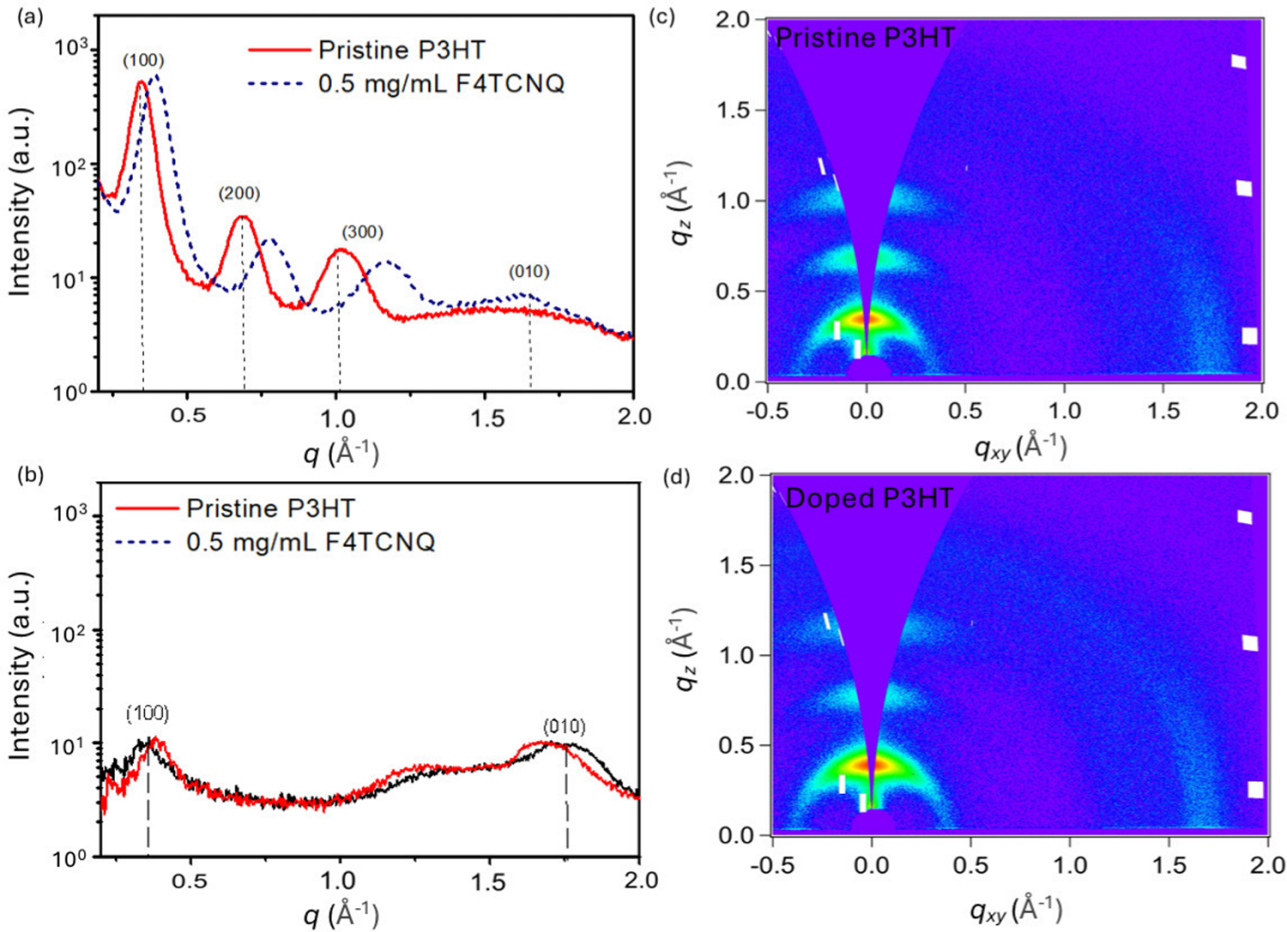
1D GIWAXS line cuts of pristine (black line) and 0.5 mg/mL F4TCNQ/AN (red line)-doped P3HT. (a) out-of-plane and (b) in-plane. 2-GIWAXS patterns of (c) pristine and (d) 0.5 mg/mL F4TCNQ/AN-doped P3HT.

Previously, Müller’s group also reported significant increases in *T*_g_ for macroscopic tensile samples in P3HT/F4TCNQ32 and P(g2T-T)/F4TCNQ or HFSI coprocessed (blended) systems,^32,33^ accompanied by an increase in the degree of π-stacking and the elastic modulus. They attributed the enhanced stiffness to an increase in π-stacking upon doping, evidenced by the emergence of out-of-plane, face-on (010) peaks and their shifts toward lower *q* values with increasing dopant concentrations.^32,33^ Here, we also studied the crystalline packing of our samples before and after doping. However, our GIWAXS results for sequentially doped edge-on P3HT films show only a slight shift in the (010) peak *q* value (Table 1), suggesting that the π-stacking of P3HT after sequential doping is not significantly altered. The calculated π-stacking distances using the (010) peak are 4.10 Å for the pristine sample and 3.92 Å for the doped sample. The intensity of the (100) peaks in the 2D GIWAXS data (Figure 6a) remains most prominent in the *q*_*z*_ direction, indicating an edge-on orientation, with no noticeable difference in the orientation between the pristine and doped samples. There is, however, a shift of the (100) lamellar peak to a lower *q* value (Table 1), which is consistent with the report on the intercalation of the F4TCNQ dopant into the lamellar region during sequential doping.^47−49^ The calculated interlamellar packing distances are 18.48 Å for the doped sample and 16.11 Å for the pristine sample. Moreover, we observed an approximate 33% increase in the relative degree of crystallinity (RDoC) when comparing the pristine and F4TCNQ-doped films, based on the integrated area of the (100) peak from the pole figure (Figure S9) as a reference (Table S6).

**Table 1:** Summary of Peak Shifts for GIWAXS Analysis of F4TCNQ-Doped and Pristine P3HT Spin-Coated Films

	*q* (Å^–1^)		
Peak Assignment	Pristine	Doped	Δ*q* (Å^–1^)^a^
(100)	0.34	0.39	0.05
(200)	0.69	0.78	0.09
(300)	1.02	1.17	0.15
(010)	1.53	1.60	0.07

a Δ*q* = *q*_Doped_ – *q*_Pristine_.

Lastly, we performed flash differential scanning calorimetry (Flash DSC) to further investigate the glass transition temperature (*T*_g_) of our materials (Figure 5b). We tested drop-cast samples from a mixed solution using the same doping/polymer concentrations as the sequential doping for relative comparison. The *T*_g_ of the pristine sample increased from 41.7 to 48.3 °C upon mixed solution doping. Due to the fast cooling rate, the observed *T*_g_ value is higher than the typical value measured via conventional DSC for P3HT samples. Even at this low doping concentration (equivalent to 1 mg/mL F4TCNQ and 10.5 mg/mL P3HT in CB), an increase of approximately 7 °C in the *T*_g_ for the doped film was observed (Figure 5b). This suggests that the slowed backbone dynamics (higher *T*_g_) and the associated higher RDoC (100) after F4TCNQ doping account for the stiffening of the doped P3ATs. Overall, our observation indicates that the efficiency of doping and the physical state of the polymer are important factors influencing the modulus of the resulting doped sample.

## Conclusions

In summary, sequential doping can influence the mechanical properties of CPs. The sensitivity of the elastic modulus to doping is highly dependent on the original state of the CPs and the doping efficiency. All the P3ATs can be efficiently doped with F4TCNQ, as supported by the appearance of polaron bands and anion peaks in the UV–vis spectra. However, only P3HT and P3OT, which have side-chain lengths of C6 or longer, show a significant enhancement in elastic modulus. This indicates that CPs in a rubbery state with a *T*_g_ below or near room temperature are more responsive to sequential doping with F4TCNQ. Conversely, low doping efficiency was observed in donor–acceptor (D–A) type CPs with high ionization energy (IE). Treatment of DPP-C_10_C_12_-T with F4TCNQ does not produce a significant amount of doping product, as shown by UV–vis spectroscopy, and its modulus remains unchanged without exhibiting a plasticizing effect. These findings deepen our understanding of the mechanics of doped CP thin films and highlight the potential for fine-tuning the mechanical properties of CP materials for specific applications in semiconducting stretchable devices through sequential molecular doping, without substantially affecting film quality or morphology.

## Experimental Section

### Materials

PDMS (Sylgard 184, Dow Corning), P3HT (Ossila, *M*w = 74 kDa, dispersity (*Đ*) = 2.1, regioregularity = 97.3%), P3HT (Rieke Metals, Mw = 30–90 kDa, Regiorandom),P3OT (Rieke Metals, Mw = 70–90 kDa, Regioregularity = 91–93%), P3BT (Rieke Metals, Mw = 50–70 kDa, Regioregularity = 80–90%) and Poly(4-styrenesulfonic acid) (PSS) solution (Sigma-Aldrich, Mw = 75 kDa) were purchased and used as received. The DPP-C_10_C_12_-T polymer (*M*_n_ = 81 kDa, dispersity (*Đ*) = 1.81) was synthesized by previously reported method.^62^ F4TCNQ (>98%) dopant was purchased from TCI America and used without further purification. FeCl_3_ (anhydrous, 98%) was obtained from Fisher Scientific. Solvents used for processing polymers and dopants such as chlorobenzene (Sigma-Aldrich, anhydrous) and acetonitrile (Oakwood chemicals, HPLC grade) were used as received.

### Preparation of Pristine and Sequentially Doped Dog-Bone CP Tensile Samples

The general procedures of tensile preparation follow previous reports.^43^ A sacrificial layer of PSS was deposited onto a plasma-treated silicon wafer by spin-coating 3 wt.% PSS in water solution at 4000 rpm. The PSS-coated Si wafer was briefly heated at 80 °C for two mins to remove moisture. Then, the CPs of interest dissolved in chlorobenzene at a specific concentration were spin-coated onto the PSS/Si surface at a designated spin rate to achieve a film thickness of 60–80 nm. The details of spin-coating parameters and resultant film thicknesses can be found in Table S5. The dried CP films were then etched into dog-bone sample geometries with a length and a width of 8 mm and 2 mm, respectively by a Ytterbium laser (λ = 1064 nm), with a 3.25 mm wide support extending from each side of the gauge to ensure a firm grip on the two edges of the tensile sample by the PDMS grips from the load cell. After establishing the dog-bone patterns on the CP/PSS/Si wafer, the pristine CP films were split into individual dog bones for mechanical testing. The doped CP tensile samples were prepared by covering the entire CP/PSS/Si wafer with a dopant/acetonitrile solution for ∼15 s and spin-coated to remove the excess solution at 2000 rpm. The resultant doped films were not rinsed by acetonitrile to avoid partial dedoping.^63,64^

### Pseudo-Free Standing Tensile Testing

The general procedures of tensile tests follow previous reports.^42,43,62^ Two aluminum grips from the load cell (Kyowa Inc.) coated with a thin layer of PDMS (≈0.5 mm) were lowered to contact the CP tensile floating on DI water. During the tensile test, the CP film was stretched at a constant displacement rate of 4 × 10^–3^ mm s^–1^ via a motorized linear stage equipped with a digital encoder, and the force exerted on the CP film was simultaneously measured at a frequency of 10 Hz by the load cell. Given that the cross-sectional area and the length of the thin films are known, the obtained force–displacement curves can be used to derive the stress–strain relationship of the CP films.

### Characterization of the Pristine and Doped CP Films

UV–vis-NIR absorption spectra were recorded on an Agilent Cary 5000 UV–vis-NIR spectrometer. For the absorption measurements of pristine and doped CP films on PSS/glass substrates (non-FOW), the CP layer was spin-coated onto PSS/glass substrates using the same spin rate in preparing its CP/PSS/Si tensile film. For the FOW samples, the CP layer from a CP/PSS/Si sample was first floated on DI water before being collected on a glass slide from the water underneath. After drying of either CP/PSS/glass or the CP tensile/glass, it was mounted on a sample holder for the UV–vis-NIR measurement.

The thickness of the CP film was determined by a DektakXT stylus profilometer (Bruker) via scanning over scratches made on a CP tensile/glass. The surface morphology of the CP tensile/Si sample was obtained by Atomic Force Microscopy (Asylum Research) in semicontact mode. The AFM images were processed by Gwyddion software (http://gwyddion.net/, Czech Metrology Institute).

An ESCALAB Xi+ spectrometer from Thermo Fisher was used to perform X-ray photoelectron spectroscopy experiments. The spectrometer was equipped with a monochromatic Al Kα X-ray source (1486.6 eV, 900 μm spot size). Spectra were obtained using the standard magnetic lens mode and charge compensation at a base pressure of 4 × 10^–7^ mbar with a take off angle of 90°. Two spots were analyzed per sample, and the reported elemental percentages represent the average. Quantification of the XPS survey scans was carried out using Avantage software from Thermo Fisher.

Grazing Incidence Wide Angle X-ray scattering of films was performed by a Xenocs Xeuss 2.0 SAXS/WAXS 2–10 m beamline equipped with a rotating GeniX 3D copper (Cu) anode X-ray source with a wavelength of 1.54 Å at 8.0 keV and 0.8 × 1.2 mm beam size, equipped with a Pilatus 1 M 2D area detector. Silver behenate was used as the calibrant and referenced to determine the sample-to-detector distance. The sample chamber was pumped down to several millibars to avoid air scattering. The sample-to-detector distance was 154 mm, and the exposure time of each sample was 1 h. Data was processed by using Igor Pro’s Nika software package.

Flash Differential Scanning Calorimetry was performed utilizing a METTLER toledo Flash DSC instrument with a temperature range of −90 to 250 °C following a heat, cool protocol, heating at 727 °C/s and a cooling rate of (4.4 °C/s). The glass transition temperature (*T*_g_) was calculated using the METTLER toledo analysis software via the Midpoint ISO method. The corresponding 1st derivative plots were also presented in Figure 5b for the verifications of *T*_g_ values.

## Supporting Information Available

XPS data, processing condition, stress relaxation, UV–vis and tensile testing for FeCl_3_-doped sample, and GPC results (PDF)

## References

[ref1] Ashizawa M.; Zheng Y.; Tran H.; Bao Z. Intrinsically stretchable conjugated polymer semiconductors in field effect transistors. Prog. Polym. Sci. 2020, 100, 101181 10.1016/j.progpolymsci.2019.101181.

[ref2] Guo X.; Baumgarten M.; Müllen K. Designing π-conjugated polymers for organic electronics. Prog. Polym. Sci. 2013, 38 (12), 1832–1908. 10.1016/j.progpolymsci.2013.09.005.

[ref3] Allard S.; Forster M.; Souharce B.; Thiem H.; Scherf U. Organic semiconductors for solution-processable field-effect transistors (OFETs). Angew. Chem., Int. Ed. 2008, 47 (22), 4070–4098. 10.1002/anie.200701920. 18357603

[ref4] Facchetti A. Semiconductors for organic transistors. materials today 2007, 10 (3), 28–37. 10.1016/S1369-7021(07)70017-2.

[ref5] Li G.; Zhu R.; Yang Y. Polymer solar cells. Nat. Photonics 2012, 6 (3), 153–161. 10.1038/nphoton.2012.11.

[ref6] Wang G.; Melkonyan F. S.; Facchetti A.; Marks T. J. All-polymer solar cells: recent progress, challenges, and prospects. Angew. Chem., Int. Ed. 2019, 58 (13), 4129–4142. 10.1002/anie.201808976. 30395372

[ref7] Sekitani T.; Someya T. Stretchable, large-area organic electronics. Adv. Mater. 2010, 22 (20), 2228–2246. 10.1002/adma.200904054. 20229571

[ref8] Wang G. N.; Gasperini A.; Bao Z. Stretchable polymer semiconductors for plastic electronics. Adv. Electron. Mater. 2018, 4 (2), 1700429 10.1002/aelm.201700429.

[ref9] Lee Y.; Zhou H.; Lee T.-W. One-dimensional conjugated polymer nanomaterials for flexible and stretchable electronics. J. Mater. Chem. C 2018, 6 (14), 3538–3550. 10.1039/C7TC05927B.

[ref10] Ocheje M. U.; Charron B. P.; Nyayachavadi A.; Rondeau-Gagné S. Stretchable electronics: Recent progress in the preparation of stretchable and self-healing semiconducting conjugated polymers. Flexible and Printed Electronics 2017, 2 (4), 043002 10.1088/2058-8585/aa9c9b.

[ref11] Lussem B.; Keum C.-M.; Kasemann D.; Naab B.; Bao Z.; Leo K. Doped organic transistors. Chem. Rev. 2016, 116 (22), 13714–13751. 10.1021/acs.chemrev.6b00329. 27696874

[ref12] Olthof S.; Mehraeen S.; Mohapatra S. K.; Barlow S.; Coropceanu V.; Brédas J.-L.; Marder S. R.; Kahn A. Ultralow doping in organic semiconductors: evidence of trap filling. Phys. Rev. Lett. 2012, 109 (17), 176601 10.1103/PhysRevLett.109.176601. 23215211

[ref13] Olthof S.; Singh S.; Mohapatra S. K.; Barlow S.; Marder S. R.; Kippelen B.; Kahn A. Passivation of trap states in unpurified and purified C60 and the influence on organic field-effect transistor performance. Appl. Phys. Lett. 2012, 101 (25), 253303 10.1063/1.4772551.

[ref14] Tietze M. L.; Pahner P.; Schmidt K.; Leo K.; Lüssem B. Doped organic semiconductors: trap-filling, impurity saturation, and reserve regimes. Adv. Funct. Mater. 2015, 25 (18), 2701–2707. 10.1002/adfm.201404549.

[ref15] Lüssem B.; Tietze M. L.; Kleemann H.; Hoßbach C.; Bartha J. W.; Zakhidov A.; Leo K. Doped organic transistors operating in the inversion and depletion regime. Nat. Commun. 2013, 4 (1), 2775 10.1038/ncomms3775. 24225722 PMC3868197

[ref16] Lashkov I.; Krechan K.; Ortstein K.; Talnack F.; Wang S.-J.; Mannsfeld S. C.; Kleemann H.; Leo K. Modulation doping for threshold voltage control in organic field-effect transistors. ACS Appl. Mater. Interfaces 2021, 13 (7), 8664–8671. 10.1021/acsami.0c22224. 33569958

[ref17] Lindorf M.; Mazzio K.; Pflaum J.; Nielsch K.; Brütting W.; Albrecht M. Organic-based thermoelectrics. J. Mater. Chem. A 2020, 8 (16), 7495–7507. 10.1039/C9TA11717B.

[ref18] Russ B.; Glaudell A.; Urban J. J.; Chabinyc M. L.; Segalman R. A. Organic thermoelectric materials for energy harvesting and temperature control. Nat. Rev. Mater. 2016, 1 (10), 1–14. 10.1038/natrevmats.2016.50.

[ref19] Massetti M.; Jiao F.; Ferguson A. J.; Zhao D.; Wijeratne K.; Würger A.; Blackburn J. L.; Crispin X.; Fabiano S. Unconventional thermoelectric materials for energy harvesting and sensing applications. Chem. Rev. 2021, 121 (20), 12465–12547. 10.1021/acs.chemrev.1c00218. 34702037

[ref20] Rivnay J.; Inal S.; Salleo A.; Owens R. M.; Berggren M.; Malliaras G. G. Organic electrochemical transistors. Nat. Rev. Mater. 2018, 3 (2), 1–14. 10.1038/natrevmats.2017.86.

[ref21] Flagg L. Q.; Bischak C. G.; Onorato J. W.; Rashid R. B.; Luscombe C. K.; Ginger D. S. Polymer crystallinity controls water uptake in glycol side-chain polymer organic electrochemical transistors. J. Am. Chem. Soc. 2019, 141 (10), 4345–4354. 10.1021/jacs.8b12640. 30779568

[ref22] Flagg L. Q.; Giridharagopal R.; Guo J.; Ginger D. S. Anion-dependent doping and charge transport in organic electrochemical transistors. Chem. Mater. 2018, 30 (15), 5380–5389. 10.1021/acs.chemmater.8b02220.

[ref23] Cendra C.; Giovannitti A.; Savva A.; Venkatraman V.; McCulloch I.; Salleo A.; Inal S.; Rivnay J. Role of the anion on the transport and structure of organic mixed conductors. Adv. Funct. Mater. 2019, 29 (5), 1807034 10.1002/adfm.201807034.

[ref24] Savva A.; Cendra C.; Giugni A.; Torre B.; Surgailis J.; Ohayon D.; Giovannitti A.; McCulloch I.; Di Fabrizio E.; Salleo A.; Rivnay J.; Inal S. Influence of water on the performance of organic electrochemical transistors. Chem. Mater. 2019, 31 (3), 927–937. 10.1021/acs.chemmater.8b04335.

[ref25] Dai Y.; Dai S.; Li N.; Li Y.; Moser M.; Strzalka J.; Prominski A.; Liu Y.; Zhang Q.; Li S.; Hu H.; Liu W.; Chatterji S.; Cheng P.; Tian B.; McCulloch I.; Xu J.; Wang S. Stretchable redox-active semiconducting polymers for high-performance organic electrochemical transistors. Adv. Mater. 2022, 34 (23), 2201178 10.1002/adma.202201178. 35448913

[ref26] Ko J.; Wu X.; Surendran A.; Muhammad B. T.; Leong W. L. Self-healable organic electrochemical transistor with high transconductance, fast response, and long-term stability. ACS Appl. Mater. Interfaces 2020, 12 (30), 33979–33988. 10.1021/acsami.0c07913. 32615752

[ref27] Rivnay J.; Leleux P.; Ferro M.; Sessolo M.; Williamson A.; Koutsouras D. A.; Khodagholy D.; Ramuz M.; Strakosas X.; Owens R. M.; Benar C.; Badier J. M.; Bernard C.; Malliaras G. G. High-performance transistors for bioelectronics through tuning of channel thickness. Sci. Adv. 2015, 1 (4), e1400251 10.1126/sciadv.1400251. 26601178 PMC4640642

[ref28] Cao Y.; Smith P.; Heeger A. J. Mechanical and electrical properties of polyacetylene films oriented by tensile drawing. Polymer 1991, 32 (7), 1210–1218. 10.1016/0032-3861(91)90223-6.

[ref29] Tokito S.; Smith P.; Heeger A. J. Highly conductive and stiff fibres of poly (2, 5-dimethoxy-p-phenylenevinylene) prepared from soluble precursor polymer. Polymer 1991, 32 (3), 464–470. 10.1016/0032-3861(91)90450-W.

[ref30] Tokito S.; Smith P.; Heeger A. J. Mechanical and electrical properties of poly-(2, 5-thienylene vinylene) fibers. Synth. Met. 1990, 36 (2), 183–194. 10.1016/0379-6779(90)90051-L.

[ref31] Craighero M.; Guo J.; Zokaei S.; Griggs S.; Tian J.; Asatryan J.; Kimpel J.; Kroon R.; Xu K.; Reparaz J. S.; Martín J.; McCulloch I.; Campoy-Quiles M.; Müller C. Impact of oligoether side-chain length on the thermoelectric properties of a polar polythiophene. ACS Appl. Electron. Mater. 2023, 6 (5), 2909–2916. 10.1021/acsaelm.3c00936. 38828039 PMC11137803

[ref32] Hultmark S.; Craighero M.; Zokaei S.; Kim D.; Järsvall E.; Farooqi F.; Marina S.; Kroon R.; Martin J.; Zozoulenko I.; Müller C. Impact of oxidation-induced ordering on the electrical and mechanical properties of a polythiophene co-processed with bistriflimidic acid. J. Mater. Chem. C 2023, 11 (24), 8091–8099. 10.1039/D2TC03927C.

[ref33] Zokaei S.; Kim D.; Järsvall E.; Fenton A. M.; Weisen A. R.; Hultmark S.; Nguyen P. H.; Matheson A. M.; Lund A.; Kroon R.; Chabinyc M. L.; Gomez E. D.; Zozoulenko I.; Müller C. Tuning of the elastic modulus of a soft polythiophene through molecular doping. Mater. Horiz. 2022, 9 (1), 433–443. 10.1039/D1MH01079D. 34787612

[ref34] Hynynen J.; Jarsvall E.; Kroon R.; Zhang Y.; Barlow S.; Marder S. R.; Kemerink M.; Lund A.; Müller C. Enhanced thermoelectric power factor of tensile drawn poly (3-hexylthiophene). ACS Macro Lett. 2019, 8 (1), 70–76. 10.1021/acsmacrolett.8b00820. 30701126 PMC6344060

[ref35] Kroon R.; Hofmann A. I.; Yu L.; Lund A.; Müller C. Thermally activated in situ doping enables solid-state processing of conducting polymers. Chem. Mater. 2019, 31 (8), 2770–2777. 10.1021/acs.chemmater.8b04895. 31303693 PMC6614883

[ref36] Mun J.; Kang J.; Zheng Y.; Luo S.; Wu Y.; Gong H.; Lai J. C.; Wu H. C.; Xue G.; Tok J. B. H. F4-TCNQ as an additive to impart stretchable semiconductors with high mobility and stability. Adv. Electron. Mater. 2020, 6 (6), 2000251 10.1002/aelm.202000251.

[ref37] Moulton J.; Smith P. Electrical and mechanical properties of oriented poly (3-alkylthiophenes) I. Doping-enhanced stiffness of poly (3-octylthiophene). Synth. Met. 1991, 40 (1), 13–22. 10.1016/0379-6779(91)91484-R.

[ref38] Moulton J.; Smith P. Electrical and mechanical properties of oriented poly (3-alkylthiophenes): 2. Effect of side-chain length. Polymer 1992, 33 (11), 2340–2347. 10.1016/0032-3861(92)90525-2.

[ref39] Paleti S. H. K.; Kim Y.; Kimpel J.; Craighero M.; Haraguchi S.; Müller C. Impact of doping on the mechanical properties of conjugated polymers. Chem. Soc. Rev. 2024, 53 (4), 1702–1729. 10.1039/D3CS00833A. 38265833 PMC10876084

[ref40] Jacobs I. E.; Aasen E. W.; Oliveira J. L.; Fonseca T. N.; Roehling J. D.; Li J.; Zhang G.; Augustine M. P.; Mascal M.; Moulé A. J. Comparison of solution-mixed and sequentially processed P3HT: F4TCNQ films: effect of doping-induced aggregation on film morphology. J. Mater. Chem. C 2016, 4 (16), 3454–3466. 10.1039/C5TC04207K.

[ref41] Scholes D. T.; Hawks S. A.; Yee P. Y.; Wu H.; Lindemuth J. R.; Tolbert S. H.; Schwartz B. J. Overcoming film quality issues for conjugated polymers doped with F4TCNQ by solution sequential processing: Hall effect, structural, and optical measurements. J. Phys. Chem. Lett. 2015, 6 (23), 4786–4793. 10.1021/acs.jpclett.5b02332. 26554820

[ref42] Zhang S.; Ocheje M. U.; Luo S.; Ehlenberg D.; Appleby B.; Weller D.; Zhou D.; Rondeau-Gagné S.; Gu X. Probing the viscoelastic property of pseudo free-standing conjugated polymeric thin films. Macromol. Rapid Commun. 2018, 39 (14), 1800092 10.1002/marc.201800092. 29749111

[ref43] Galuska L. A.; Muckley E. S.; Cao Z.; Ehlenberg D. F.; Qian Z.; Zhang S.; Rondeau-Gagné S.; Phan M. D.; Ankner J. F.; Ivanov I. N.; Gu X. SMART transfer method to directly compare the mechanical response of water-supported and free-standing ultrathin polymeric films. Nat. Commun. 2021, 12 (1), 2347 10.1038/s41467-021-22473-w. 33879775 PMC8058343

[ref44] Kim H. J.; Kim J.-H.; Ryu J.-H.; Kim Y.; Kang H.; Lee W. B.; Kim T.-S.; Kim B. J. Architectural engineering of rod–coil compatibilizers for producing mechanically and thermally stable polymer solar cells. ACS Nano 2014, 8 (10), 10461–10470. 10.1021/nn503823z. 25256674

[ref45] Liu Y.; Chen Y.-C.; Hutchens S.; Lawrence J.; Emrick T.; Crosby A. J. Directly measuring the complete stress–strain response of ultrathin polymer films. Macromolecules 2015, 48 (18), 6534–6540. 10.1021/acs.macromol.5b01473.

[ref46] Zhang S.; Galuska L. A.; Gu X. Water-assisted mechanical testing of polymeric thin-films. J. Polym. Sci. 2022, 60 (7), 1108–1129. 10.1002/pol.20210281.

[ref47] Scholes D. T.; Yee P. Y.; Lindemuth J. R.; Kang H.; Onorato J.; Ghosh R.; Luscombe C. K.; Spano F. C.; Tolbert S. H.; Schwartz B. J. The effects of crystallinity on charge transport and the structure of sequentially processed F4TCNQ-doped conjugated polymer films. Adv. Funct. Mater. 2017, 27 (44), 1702654 10.1002/adfm.201702654.

[ref48] Lim E.; Peterson K. A.; Su G. M.; Chabinyc M. L. Thermoelectric properties of poly (3-hexylthiophene)(P3HT) doped with 2, 3, 5, 6-Tetrafluoro-7, 7, 8, 8-tetracyanoquinodimethane (F4TCNQ) by vapor-phase infiltration. Chem. Mater. 2018, 30 (3), 998–1010. 10.1021/acs.chemmater.7b04849.

[ref49] Untilova V.; Zeng H.; Durand P.; Herrmann L.; Leclerc N.; Brinkmann M. Intercalation and ordering of F6TCNNQ and F4TCNQ dopants in regioregular poly (3-hexylthiophene) crystals: impact on anisotropic thermoelectric properties of oriented thin films. Macromolecules 2021, 54 (13), 6073–6084. 10.1021/acs.macromol.1c00554.

[ref50] Xin H.; Subramaniyan S.; Kwon T.-W.; Shoaee S.; Durrant J. R.; Jenekhe S. A. Enhanced open circuit voltage and efficiency of donor–acceptor copolymer solar cells by using indene-C60 bisadduct. Chem. Mater. 2012, 24 (11), 1995–2001. 10.1021/cm300355e.

[ref51] Gao W.; Kahn A. Controlled p-doping of zinc phthalocyanine by coevaporation with tetrafluorotetracyanoquinodimethane: A direct and inverse photoemission study. Appl. Phys. Lett. 2001, 79 (24), 4040–4042. 10.1063/1.1424067.

[ref52] Bijleveld J. C.; Verstrijden R. M.; Wienk M. M.; Janssen R. A. Copolymers of diketopyrrolopyrrole and thienothiophene for photovoltaic cells. J. Mater. Chem. 2011, 21 (25), 9224–9231. 10.1039/c1jm10961h.

[ref53] Tang K.; Huang L.; Lim J.; Zaveri T.; Azoulay J. D.; Guo S. Chemical doping of well-dispersed P3HT thin-film nanowire networks. ACS Applied Polymer Materials 2019, 1 (11), 2943–2950. 10.1021/acsapm.9b00653.

[ref54] Pingel P.; Neher D. Comprehensive picture of p-type doping of P3HT with the molecular acceptor F 4 TCNQ. Phys. Rev. B: Condens. Matter Mater. Phys. 2013, 87 (11), 115209 10.1103/PhysRevB.87.115209.

[ref55] Wang C.; Duong D. T.; Vandewal K.; Rivnay J.; Salleo A. Optical measurement of doping efficiency in poly (3-hexylthiophene) solutions and thin films. Phys. Rev. B 2015, 91 (8), 085205 10.1103/PhysRevB.91.085205.

[ref56] Watts K. E.; Clary K. E.; Lichtenberger D. L.; Pemberton J. E. FTIR spectroelectrochemistry of F4TCNQ reduction products and their protonated forms. Anal. Chem. 2020, 92 (10), 7154–7161. 10.1021/acs.analchem.0c00615. 32357003

[ref57] Ma G.; Zhang S.; Galuska L. A.; Gu X. Rapid stress relaxation of high-Tg conjugated polymeric thin films. J. Polym. Sci. 2024, 62 (16), 3839–3847. 10.1002/pol.20230671.

[ref58] Qian Z.; Cao Z.; Galuska L.; Zhang S.; Xu J.; Gu X. Glass transition phenomenon for conjugated polymers. Macromol. Chem. Phys. 2019, 220 (11), 1900062 10.1002/macp.201900062.

[ref59] Dubey A.; Adhikari N.; Venkatesan S.; Gu S.; Khatiwada D.; Wang Q.; Mohammad L.; Kumar M.; Qiao Q. Solution processed pristine PDPP3T polymer as hole transport layer for efficient perovskite solar cells with slower degradation. Sol. Energy Mater. Sol. Cells 2016, 145, 193–199. 10.1016/j.solmat.2015.10.008. PMC5063754 27761487

[ref60] Karpov Y.; Erdmann T.; Stamm M.; Lappan U.; Guskova O.; Malanin M.; Raguzin I.; Beryozkina T.; Bakulev V.; Günther F.; Gemming S.; Seifert G.; Hambsch M.; Mannsfeld S.; Voit B.; Kiriy A. Molecular doping of a high mobility diketopyrrolopyrrole–dithienylthieno [3, 2-b] thiophene donor–acceptor copolymer with F6TCNNQ. Macromolecules 2017, 50 (3), 914–926. 10.1021/acs.macromol.6b02452.

[ref61] Karpov Y.; Erdmann T.; Raguzin I.; Al-Hussein M.; Binner M.; Lappan U.; Stamm M.; Gerasimov K. L.; Beryozkina T.; Bakulev V.; Anokhin D. V.; Ivanov D. A.; Günther F.; Gemming S.; Seifert G.; Voit B.; Di Pietro R.; Kiriy A. High conductivity in molecularly p-doped diketopyrrolopyrrole-based polymer: the impact of a high dopant strength and good structural order. Adv. Mater. 2016, 28 (28), 6003–6010. 10.1002/adma.201506295. 27172371

[ref62] Zhang S.; Ocheje M. U.; Huang L.; Galuska L.; Cao Z.; Luo S.; Cheng Y.; Ehlenberg D.; Goodman R. B.; Zhou D.; Liu Y.; Chiu Y.; Azoulay J. D.; Rondeau-Gagné S.; Gu X. The critical role of electron-donating thiophene groups on the mechanical and thermal properties of donor–acceptor semiconducting polymers. Adv. Electron. Mater. 2019, 5 (5), 1800899 10.1002/aelm.201800899.

[ref63] Tang K.; McFarland F. M.; Travis S.; Lim J.; Azoulay J. D.; Guo S. Aggregation of P3HT as a preferred pathway for its chemical doping with F 4-TCNQ. Chem. Commun. 2018, 54 (84), 11925–11928. 10.1039/C8CC05472J. 30283920

[ref64] Untilova V.; Biskup T.; Biniek L.; Vijayakumar V.; Brinkmann M. Control of chain alignment and crystallization helps enhance charge conductivities and thermoelectric power factors in sequentially doped P3HT: F4TCNQ films. Macromolecules 2020, 53 (7), 2441–2453. 10.1021/acs.macromol.9b02389.

