# Supplementary material for: Impact of Sequential Chemical Doping on the Thin Film Mechanical Properties of Conjugated Polymers
Source: Chem Mater. 2025 Jan 9;37(2):756–65. doi: 10.1021/acs.chemmater.4c03120 (PMC11780729; doi:10.1021/acs.chemmater.4c03120)
Supplement: Supplementary file 1 — cm4c03120_si_001.pdf [file cm4c03120_si_001.pdf]

Supporting information

Impact of Sequential Chemical Doping on the Thin Film Mechanical Properties of Conjugated Polymers

Kan Tang,[†] *Alyssa Shaw,*[†] *Saroj Upreti,*[†] *Haoyu Zhao,*[†] *Yunfei Wang,*[†] *Gage T. Mason, Jeffery Aguinaga,*[†] *Keyi Guo^, Derek Patton,*[†] *Derya Baran*[‡]*, Simon Rondeau-Gagne,*[§] *Xiaodan Gu**[†]

[†]Center for Optoelectronic Materials and Devices, School of Polymer Science and Engineering, The University of Southern Mississippi, Hattiesburg, Mississippi 39406, United States

[‡] Materials Science and Engineering Program (MSE), Physical Sciences and Engineering Division (PSE), King Abdullah University of Science and Technology, Thuwal 23955, Saudi Arabia

[§]Department of Chemistry and Biochemistry, University of Windsor, Windsor, ON N9B3P4, Canada

^Oak Grove High School, Oak Grove, Hattiesburg, Mississippi, 39402

Contents

1. XPS results for pristine and doped film-on-water CP samples 3
2. Detailed processing conditions for the spin-coating of CP samples......................................5
3. Stress relaxation experiments on pristine and doped samples..7
4. Tensile test results for F4TCNQ doped regiorandom (rra) P3HT..7
5. UV-vis results for $FeCl_3$ doped CPs...8
6. Tensile test results for $FeCl_3$-doped CPs...9
7. Additional AFM images 10
9. Gel permeation chromatogram (GPC) of the DPP-$C_{10}C_{12}$-T polymer 12
10. Additional discussion on the $FeCl_3$ doped CPs 12

1. XPS results for pristine and doped film-on-water CP samples

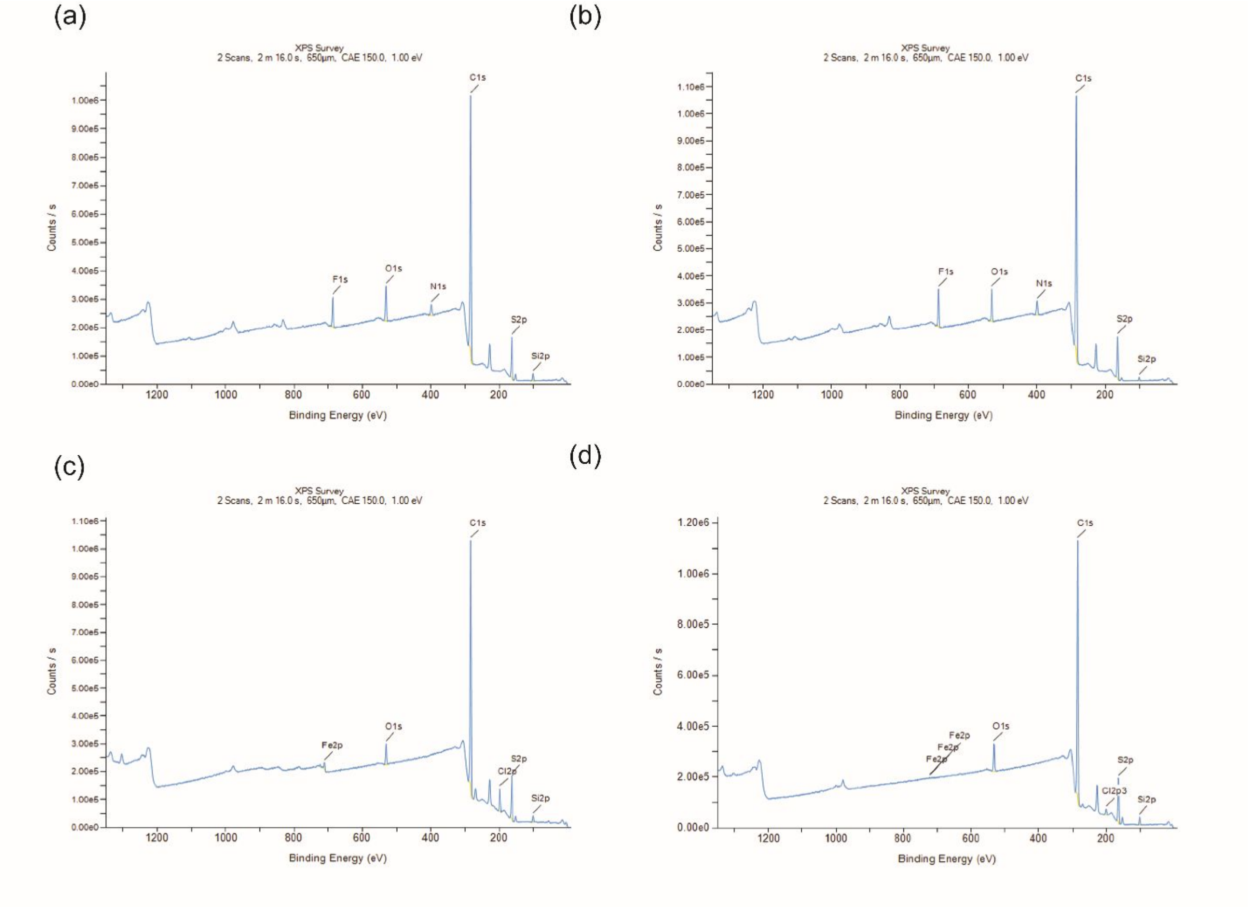

Figure S1. XPS survey spectra of sequentially doped P3HT on Si wafer before and after immersion in DI water for 10 minutes. (a) P3HT film doped with F4TCNQ/AN (0.5 mg/mL), before immersion. (b) P3HT film doped with F4TCNQ/AN (0.5 mg/mL), after immersion. (c) P3HT film doped with $FeCl_3$/AN (10 mg/mL), before immersion. (d) P3HT film doped with $FeCl_3$/AN (10 mg/mL), after immersion. P3HT dissolved in chlorobenzene (15 mg/mL) was spun-coated on Si at 900 rpm. Sequential doping was performed by casting the dopant solution on top of the P3HT layer, followed by the spin-off of excess solution at 2000 rpm.

Table S1. Summary of XPS survey on the major elements presented in F4TCNQ doped P3HT on Si wafer before immersion in DI water.

P3HT (F4TCNQ doped)/Si, before immersion				
Peak Name	Peak BE (eV)	FWHM (eV)	Area	Atomic %
C_{1s}	284.13	3.53	3426698.98	80.70
S_{2p}	163.74	3.68	550838.26	6.52
O_{1s}	531.41	3.75	491039.28	4.60
F_{1s}	687.18	3.46	400500.67	2.92
N_{1s}	399.01	4.79	206858.66	3.08
Si_{2p}	101.22	3.36	90039.16	2.17

Table S2. Summary of XPS survey on the major elements presented in F4TCNQ doped P3HT on Si wafer after immersion in DI water for 10 minutes.

P3HT (F4TCNQ doped)/Si, after immersion				
Peak Name	Peak BE (eV)	FWHM (eV)	Area	Atomic %
C_{1s}	284.22	3.59	3652311.29	81.16
S_{2p}	163.8	3.65	579573.61	6.47
F_{1s}	687.29	3.56	535362.14	3.68
O_{1s}	531.9	3.75	488833.45	4.32
N_{1s}	399.18	4.31	238457.51	3.35
Si_{2p}	101.63	3.32	44146.41	1.00

Table S3. Summary of XPS survey on the major elements presented in $FeCl_3$ doped P3HT on Si wafer before immersion in DI water.

P3HT ($FeCl_3$ doped)/Si, before immersion

Peak Name	Peak BE (eV)	FWHM (eV)	Area	Atomic %
C_{1s}	284	3.48	3232479.68	83.89
S_{2p}	163.76	3.76	607460.16	7.93
Cl_{2p}	198.29	3.95	325605.44	2.96
O_{1s}	531.11	3.45	274188.56	2.83
Si_{2p}	101.07	3.39	79155.88	2.10
Fe_{2p}	710.7	3.7	123923.82	0.29

Table S4. Summary of XPS survey on the major elements presented in $FeCl_3$ doped P3HT on Si wafer after immersion in DI water for 10 minutes.

P3HT ($FeCl_3$ doped)/Si, after immersion				
Peak Name	Peak BE (eV)	FWHM (eV)	Area	Atomic %
C_{1s}	284.13	3.35	3516349.03	84.46
S_{2p}	163.66	3.59	646242.29	7.80
O_{1s}	531.71	3.47	407296.91	3.89
Si_{2p}	101.64	3.3	103567.47	2.54
Cl_{2p}	200.01	4.32	95595.99	1.24
Fe_{2p}	722.62	0.09	24133.78	0.05

2. Detailed processing conditions for the spin-coating of CP samples

Table S5. Processing parameters of each spun-coated CP thin film with its corresponding film thickness and surface roughness (RMS) values

Entry	CP/dopant	CP Concentration (mg/ml)	Spin rate for CP (rpm)[a]	Thickness[b] (nm)	RMS[c] (nm)
1	P3HT	15	900	81 ± 2.0	6
2	P3HT w 0.1 mg/mL F4TCNQ	15	900	82 ± 1.0	6
3	P3HT w 0.3 mg/mL F4TCNQ	15	900	86 ± 2.0	6
4	P3HT w 0.5 mg/mL F4TCNQ	15	900	82 ± 1.5	6
5	P3HT w 0.09 mg/mL $FeCl_3$	15	900	86 ± 1.0	5
6	P3HT w 0.18 mg/mL $FeCl_3$	15	900	81 ± 2.6	5.5
7	P3HT w 0.36 mg/mL $FeCl_3$	15	900	80 ± 2.2	5.5
8	P3OT	15	1000	65 ± 1.5	0.6
9	P3OT w 0.5 mg/mL F4TCNQ	15	1000	65 ± 2.4	0.6
10	P3OT w 0.36 mg/mL $FeCl_3$	15	1000	66 ± 2.0	0.6
11	P3BT	10	500	73 ± 2.8	1.5

12	P3BT w 0.5 mg/mL F4TCNQ	10	500	80 ± 2.3	1.7
13	P3BT w 0.36 mg/mL $FeCl_3$	10	500	77 ± 2.0	1.4
14	DPP-$C_{10}C_{12}$-T	13.3	800	76 ± 1.6	1.7
15	DPP-$C_{10}C_{12}$-T w 0.5 mg/mL F4TCNQ	13.3	800	84 ± 3.5	1.1

[a] The spin rate for the dopant in acetonitrile is always 2000 rpm. [b] Film thickness was determined by a profilometer. [c] RMS values were obtained by the analysis of AFM images.

3. Stress relaxation experiments on pristine and doped samples

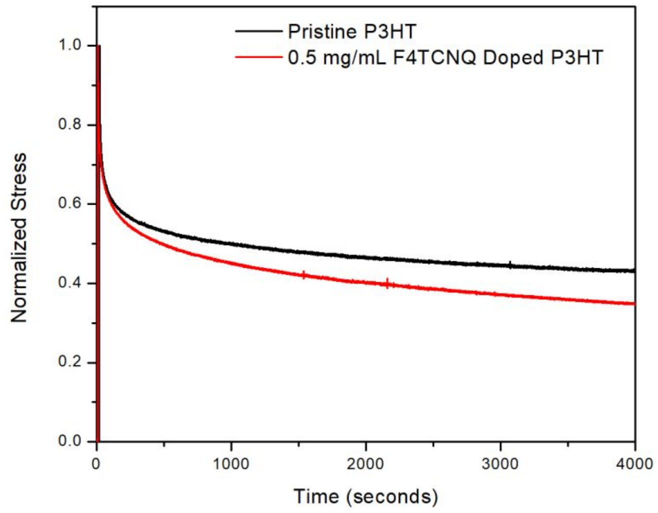

Figure S2. Time-dependent stress of pristine P3HT film and P3HT film doped with 0.5 mg/mL F4TCNQ/AN under low strain (~ 5%) to avoid yielding of the samples.

4. Tensile test results for F4TCNQ doped regiorandom (rra) P3HT

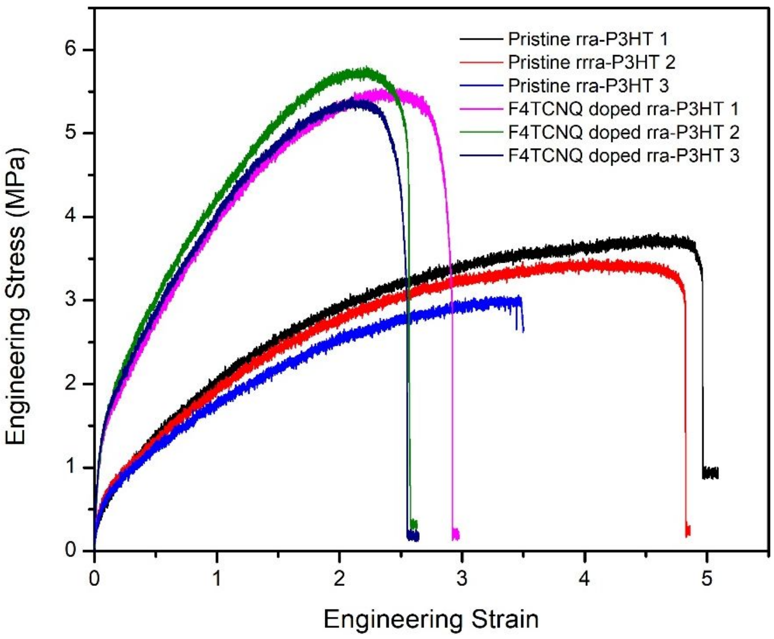

Figure S3. Stress-strain curves of pristine and sequentially doped (F4TCNQ/AN) rra-P3HT using pseudo-free-standing tensile test. Each test was reproduced 3 times. The preparation of the pristine and doped rra-P3HT films follows the general tensile protocol. Conditions: 15 mg/mL rra-P3HT in chlorobenzene, spin rate 900 rpm, 0.5 mg/mL of F4TCNQ/AN for sequential doping. The thicknesses of the films are 86-87 nm.

5. UV-vis results for $FeCl_3$ doped CPs

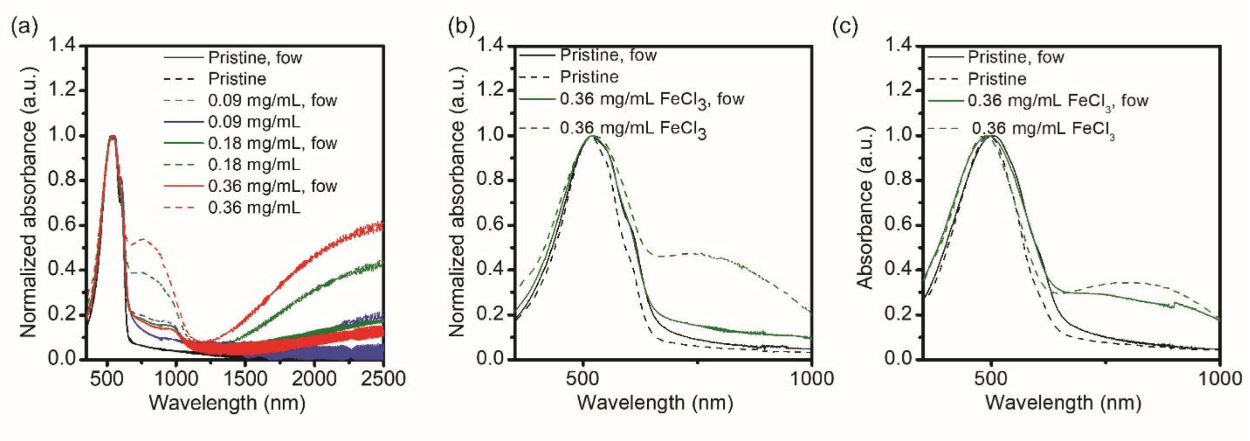

Figure S4. The UV-vis spectra of pristine and sequentially doped P3HT, P3OT, and P3BT thin films by $FeCl_3$ in acetonitrile. The dashed lines represent spectra of P3ATs/PSS/glass samples, and the solid lines are associated with film-on-water samples. Note that 0.36 mg/mL of $FeCl_3$/AN equals 1.1 mM.

6. Tensile test results for $FeCl_3$-doped CPs

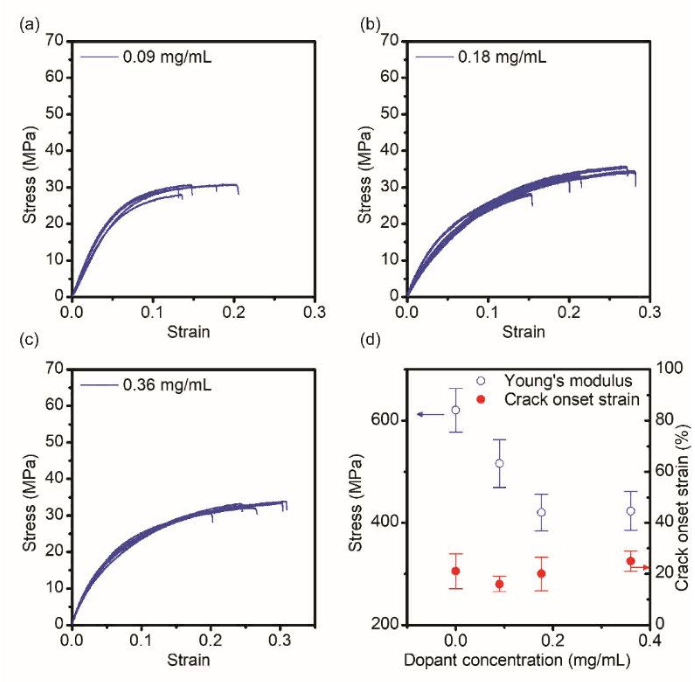

Figure S5. Stress-strain curves of pristine and sequentially doped P3HT using pseudo-free-standing tensile test. (a) P3HT doped with 0.09 mg/mL $FeCl_3$/AN. (b) P3HT doped with 0.18 mg/mL $FeCl_3$/AN. (c) P3HT doped with 0.36 mg/mL $FeCl_3$/AN. (d) Dopant concentration dependence on the stress and strain of the pristine and doped P3HT.

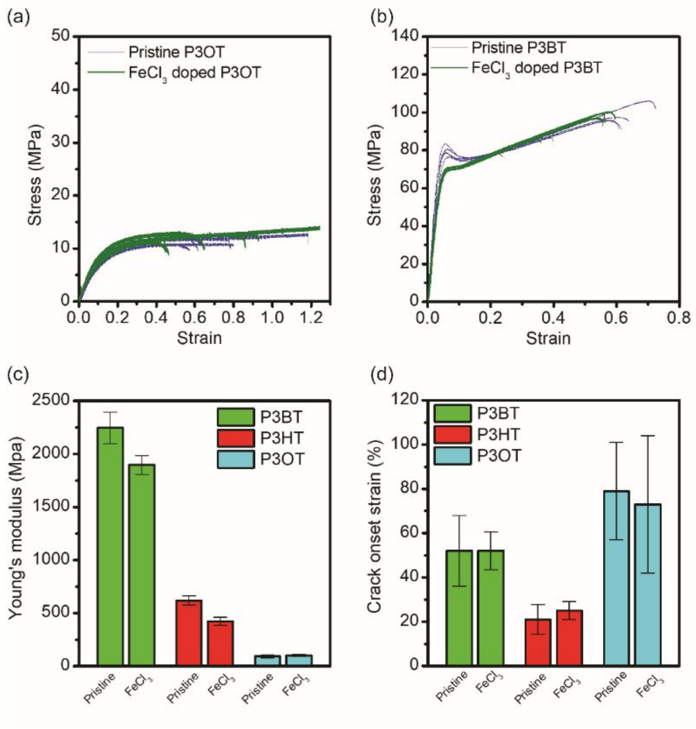

Figure S6. Stress-strain curves of pristine and sequentially doped (a) P3OT and (b) P3BT by $FeCl_3$/AN using pseudo-free-standing tensile test. Note that the $FeCl_3$/AN concentration is 0.36 mg/mL (1.1 mM).

7. Additional AFM images

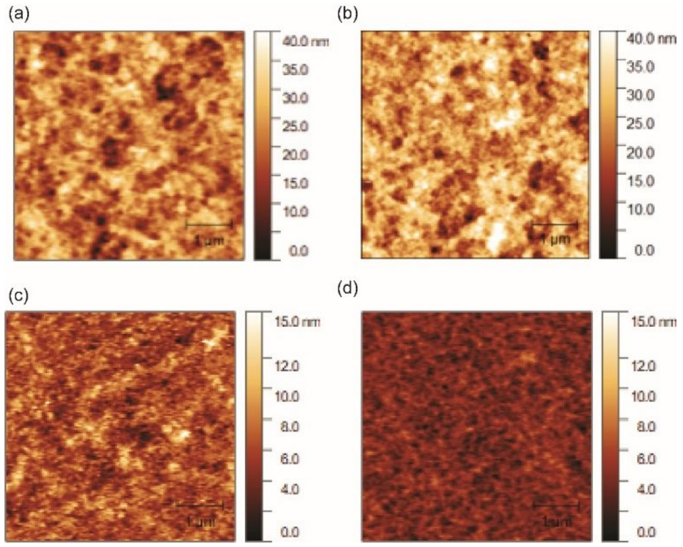

Figure S7. AFM images of pristine and F4TCNQ doped CPs. (a) P3HT doped with 0.1 mg/mL F4TCNQ/AN. (b) P3HT doped with 0.3 mg/mL F4TCNQ/AN. (c) Pristine DPP-$C_{10}C_{12}$-T. (d) DPP-$C_{10}C_{12}$-T doped with 0.5 mg/m F4TCNQ/AN. All images are in a size of 5 × 5 μm square.

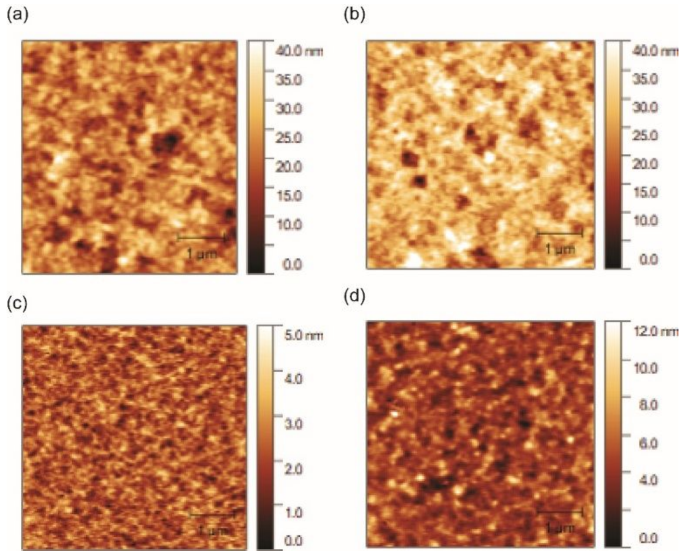

Figure S8. AFM images of pristine and $FeCl_3$ doped CPs. (a) P3HT doped with 0.09 mg/mL $FeCl_3$/AN. (b) P3HT doped with 0.18 mg/mL $FeCl_3$/AN. (c) P3OT doped with 0.36 mg/mL $FeCl_3$/AN. (d) P3BT doped with 0.36 mg/mL $FeCl_3$/AN. All images are in a size of 5 × 5 μm square.

8. Pole plot and relative degree of crystallinity (RDoC) calculation

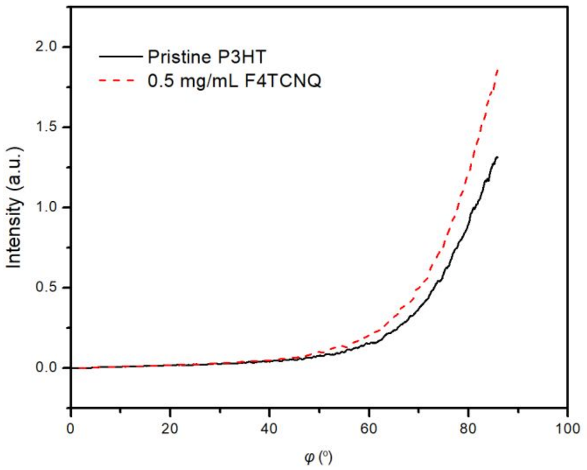

Figure S9. Pole plot for RDoC calculation of F4TCNQ/AN treated P3HT (red line) and pristine P3HT (black line).

Table S6. Summary of integration (area) in the pole figure (Figure S7) and calculated RDoC values of F4TCNQ doped and pristine P3HT spun coated films.

Sample	Area	RDoC
Pristine P3HT	605.74	1
Doped with 0.5 mg/mL F4TCNQ	807.87	1.33

9. Gel permeation chromatogram (GPC) of the DPP-$C_{10}C_{12}$-T polymer

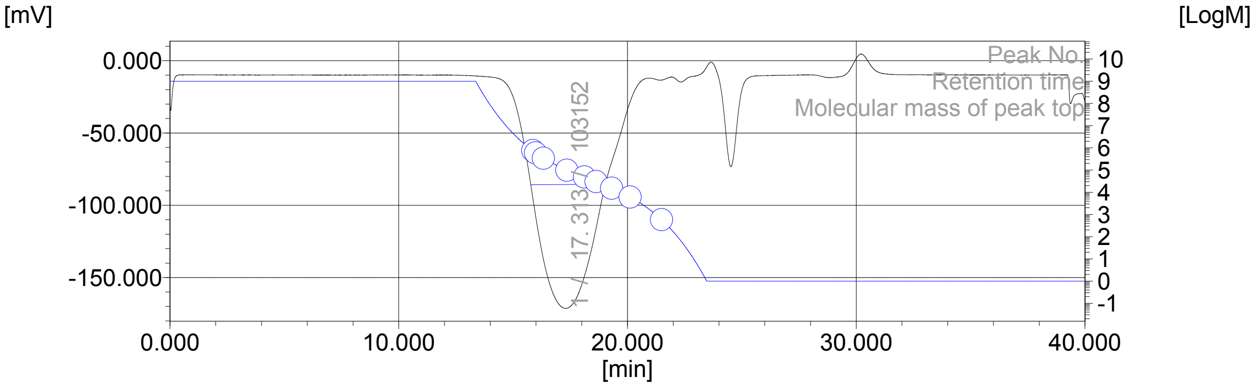

Figure S10. GPC trace of the DPP-$C_{10}C_{12}$-T polymer. The integrations for determining Mn and Mw were taken from full-width half max (FWHM).

10. Additional discussion on the $FeCl_3$ doped CPs

No plasticizing effect after F4TCNQ doping/treatment of CPs associated with lower modulus or higher ductility is observed regardless of whether the doping is efficient. Since the F4TCNQ dopant and its anion are hydrophobic, $FeCl_3$ might be a good complementary candidate to probe the plasticizing effect as it is fully water-soluble if it is energetically favored. The standard

reduction potential of $E^0(Fe^{3+}/Fe^{2+})$ is about 0.37 V[60] higher than the ferrocenium/ferrocene couple $E^0(Fc^+/Fc)$, consequently, its EA can be estimated as -5.17 eV *v.s.* vacuum level given that the Fc^+/Fc couple has an energy level of -4.8 V against the vacuum level.[61] This means that the doping of P3ATs by $FeCl_3$ should be almost as energetically favorable as the doping of P3ATs films by F4TCNQ (EA = -5.2 eV). Indeed, all three P3ATs/PSS/glass samples show characteristic polaron bands from 700 to 1000 nm (Figure S2), which indicates the successful doping of P3ATs by $FeCl_3$. Comparing the UV-vis spectra of $FeCl_3$ doped P3ATs FOW samples (solid lines, Figure S2) with the spectra of P3ATs/PSS/glass samples (dashed lines, Figure S2), a dramatic decay of the polaron bands of $FeCl_3$ doped P3ATs, which suggests the dissolution of the $FeCl_3$ and the occurrence of dedoping, is observed. The dedoping of $FeCl_3$ doped P3HT is also confirmed by XPS, which shows a major loss of Fe and Cl elements after floating the film on water for 10 mins (Figure S1c and S1d, Table S3 and S4). In contrast to the doping by F4TCNQ where the modulus is enhanced with no significant leaching or dedoping of dopant, a 30% and 17% loss in the elastic modulus is found in $FeCl_3$-doped P3HT and P3BT FOW samples (Figure S3 and S4), respectively. It is also worth noting that the change of film thickness (Table S5) or surface morphology (Figure S5 and S6) before and after moderate sequential doping (~1 mM) in this work is minimal regardless of CP or dopant selection. Typically, a sequentially doped FOW sample has a larger thickness by a few nm than its pristine form, which renders the contribution from swelling a minor effect in this FOW tensile testing. Therefore, the observed modulation of mechanical properties can be mostly attributed to doping-induced phenomena. In the $FeCl_3$ doping cases, the observed plasticizing

effect might be attributed to the generation of cavities within the films after the dissolution of the $FeCl_3$ dopant away from the CP matrix, as $FeCl_3$ is a water-soluble dopant, unlike F4TCNQ.
